# Supplementary material for: Quantifying viral pandemic potential from experimental transmission studies
Source: PLoS Comput Biol. 2025 Dec 17;21(12):e1013808. doi: 10.1371/journal.pcbi.1013808 (PMC12721528; doi:10.1371/journal.pcbi.1013808)
Supplement: S1 Text — (PDF) [file pcbi.1013808.s001.pdf]

Supplementary material: S1 Text

Quantifying viral pandemic potential from experimental transmission studies

Elizabeth D. Somsen[1,*], Kayla M. Septer[2], Cassandra J. Field[2], Devanshi R. Patel[2], Anice C. Lowen [3,4], Troy C. Sutton[2,4], Katia Koelle[4,5]

1 Graduate Program in Population Biology, Ecology, and Evolution, Emory University, Atlanta, GA, USA
2 Department of Veterinary and Biomedical Sciences, The Pennsylvania State University, State College, PA, USA
3 Department of Microbiology and Immunology, Emory University, Atlanta, GA, USA
4 Emory Center of Excellence for Influenza Research and Response (Emory CEIRR), Atlanta, GA, USA
5 Department of Biology, Emory University, Atlanta, GA, USA
*for correspondence, esomsen@emory.edu

Statistical assessment of the impact of index inoculum dose on the viral kinetics in contact animals

We statistically analyzed whether the inoculum dose received by index animals affected viral kinetics in their paired contact animals. Specifically, we fit a linear regression model with index inoculum dose as predictor variable and two different features of within-host dynamics in the contact as response variables: the peak viral titer and the duration of infection. Quantification of peak viral titers in the contact animals was straightforward. Quantification of the duration of infection was straightforward in most contact animals. However, three contact animals had transiently positive viral titers either at the beginning or towards the end of their infections. The viral kinetics of these contact ferrets and their corresponding index animals are shown in Figure A. We therefore performed two separate analyses using the duration of infection of contact animals as the response variable: one where the duration of infection included the time point of the transiently positive viral titer measurement and one where the duration of infection excluded the time point of the transiently positive viral titer measurement. Exclusion of the transiently positive time point resulted in a reduction in the duration of infection for all three contact animals.

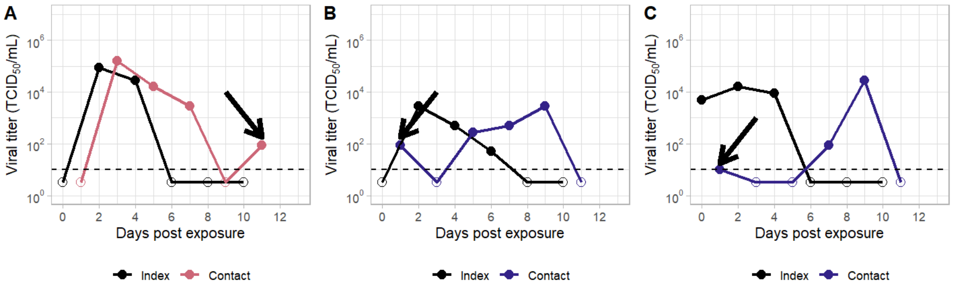

Figure A. Transmission pairs with contact animals that had transiently positive viral titer measurements. (A) Viral kinetics in a Cal/2009-inoculated index and corresponding contact. (The index animal was challenged with a 10^2 $TCID_{50}$ inoculum dose.) (B) Viral kinetics in a Hong Kong/1968-inoculated index and corresponding contact. (The index animal was challenged with a 10^2 $TCID_{50}$ inoculum dose.) (C) Viral kinetics in a Hong Kong/1968-inoculated index and corresponding contact. (The index animal was challenged with a 10^6 $TCID_{50}$ inoculum dose.) Arrows in panels (A)-(C) point at the time points at which viral titers are transiently positive.

The linear regressions on index inoculum dose are shown in Figure B. For Cal/2009, there was not a significant positive correlation between inoculum dose and either response variable. For Hong Kong/1968, we did find a significant positive correlation between inoculum dose and peak viral titers of the contact, driven mainly by the low peak titers in the 10^1 $TCID_{50}$ inoculum dose group. However, we found little to no positive correlation between inoculum dose and the duration of infection of the contact (Figure B). We therefore conclude that there is very limited statistical support for index inoculum doses impacting viral kinetic features in corresponding contact animals.

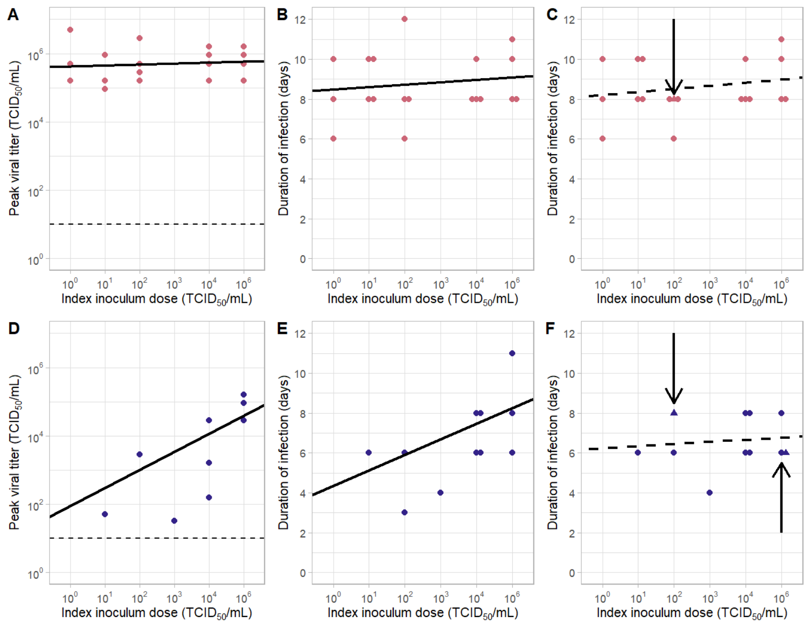

Figure B. Key metrics of viral kinetics in contact animals. In all panels, black lines show linear regressions. (A) Peak viral titers in contact animals for Cal/2009 infected animals. A statistically positive relationship between peak viral titers and index inoculum dose is not found (p = 0.62). (B) Duration of infection of contact animals for Cal/2009 infected animals. A statistically positive relationship between duration of infection and index inoculum dose is not found (p = 0.49). (C) Duration of infection of contact animals for Cal/2009 infected animals, excluding the transiently positive viral titer measurements shown in Figure A. A statistically positive relationship between duration of infection and index inoculum dose is not found (p = 0.30). (D) Peak viral titer for Hong Kong/1968 infected animals. A statistically positive relationship between peak viral titers and index inoculum dose is found (p = 0.01). (E) Duration of infection for Hong Kong/1968 infected animals. A statistically positive relationship between duration of infection and index inoculum dose is found (p = 0.04). (F) Duration of infection of contact animals for Hong Kong/1968 infected animals,excluding the transiently positive viral titer measurements shown in Figure A. A statistically positive relationship between duration of infection and index inoculum dose is not found (p = 0.68).

Parameter estimates for the force-of-infection functions

The transiently positive viral titer measurements shown in Figure A impact not only the duration of infection calculated for these two contact animals but also their times of infection (T_1 and T_2). As such, whether we consider these transiently positive viral titer measurements to be the start of infection or not will impact our estimation of the parameters in the force-of-infection functions we evaluate. In the main manuscript, we estimate these parameters assuming that the transiently-positive viral titers observed in Figure A indeed indicate the start of viral infection in these two contact animals. Table A displays the maximum likelihood estimates for parameters of all 4 functional forms under this assumption.

Functional Form	Parameters	Cal/2009 MLE	Hong Kong/1968 MLE
log_{10}	s	$s = 0.111$	$s = 0.047$
linear	s_L	$s_L = 1.60 \times 10^{-6}$	s_L =1.99 $\times 10^{-5}$
threshold	h, r	$r = 0.71, h = 3.83$	$r = 0.12, h = 1.0$
Hill	q, k_a, n	$q = 0.7, k_a = 3.8, n = 198.8$	$q = 0.3, k_a = 6000, n = 0.012$

Table A. Maximum likelihood estimates for the force-of-infection model parameters for each of the four functional forms considered (see Figure 3 in main text).

Parameter estimates for the force-of-infection functions when excluding transiently positive viral titer measurements in the contact animals

Because two of the Hong Kong/1968 contact animals have transiently positive viral titers that impact their timing of infection, we here conduct a sensitivity analysis to determine the extent to which the parameter estimates of the four functional forms change when instead considering the titers at these timepoints to be below the level of detection. This delays both T_1 and T_2 in these contact animals. Table B shows parameter estimates for the four force-of-infection functional forms evaluated (log_{10}, linear, threshold, and Hill). We only present Hong Kong/1968 results because both contact animals with transiently positive viral titers at the beginning of infection are from the Hong Kong/1968 study. The results for Cal/2009 were identical to those presented in the main text. The maximum likelihood parameter estimates for all four functional forms, shown in Figure C, are quantitatively similar to those previously estimated and shown in Table 1.

Table B further lists AICc values for each of the four functional forms to allow for model comparison when the T_1 and T_2 are modified for the two contact animals shown in Figure A (panels B,C) to reflect removal of the transiently positive viral titers. We find that the threshold model retains the best AICc score and that all scores are very similar to those presented in the main text. There are still only minor differences between the AICc scores for the threshold, Hill, and log_{10} forms, indicating that there is not strong statistical support for distinguishing between these models, even when the transiently positive tests shown in Figure A are reclassified as below the limit of detection.

Functional Form	Parameters	Hong Kong/1968 MLE	Hong Kong/1968 AICc
log_{10}	s	$s = 0.045$	65.55 (3.89)
linear	s_L	s_L =1.98 $\times 10^{-5}$	115.16 (53.49)
threshold	h, r	$r = 0.11, h = 1.0$	**61.66**
Hill	q, k_a, n	$q = 0.3, k_a = 6000, n = 0.061$	64.6 (2.94)

Table B. Maximum likelihood estimates and model selection for the force-of-infection model parameters when the transiently positive viral titers are excluded. Only results for Hong Kong/1968 are presented because there were no changes in the estimates for Cal/2009.

In Figure C, we reproduce the plots shown in Figure 3 of the main text, assuming that the transiently positive viral titers in the two contact animals shown in Figure A (panels B,C) did not indicate the start of infection. We find that the results are very similar to those shown in the main text.

Quantification of parameters at the transmission event for alternative force-of-infection functional forms

In the main text, we showed our estimates for transmission event parameters (secondary cases and generation interval) assuming a log_{10} functional form for the force of infection:

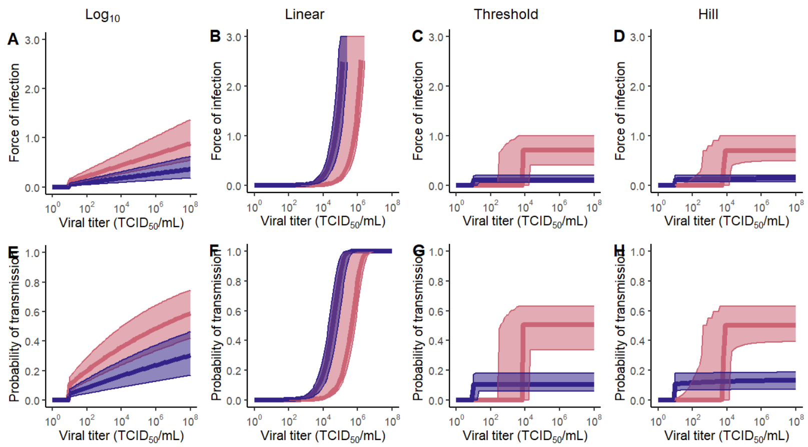

Figure C. Statistical estimation of different force-of-infection functional forms when the transiently positive viral titers in the contact animals shown in Figure 1 (panels B,C) are reclassified as falling below the limit of detection. (A)-(D) Estimated forces of infection (λ values) across a range of viral titers. (E)-(H) Probabilities of transmission given a one-day exposure to a constant viral titer, given the λ estimates from (A)-(D), respectively. Columns correspond to the different force-of-infection functional forms: (A, E) log_{10}-transformed, (B, F) linear, (C, G) threshold, and (D, H) Hill function. Table B provides maximum likelihood estimates for the force-of-infection model parameters for each of the four functional forms considered. Shaded regions show 95% confidence intervals.

$\lambda(t) = s \times log_{10}(V(t))$. Here, we present complementary results for the linear, threshold, and Hill functional forms. Relative to the log_{10} form, the linear form (Figure D) estimates a lower R_0 for both Cal/2009 and Hong Kong/1968. Estimated transmission heterogeneity is also substantially higher. Mean generation time is similar, but with a lower coefficient of variation. Transmission event parameters for the threshold functional form (Figure E) are similar to those of the log_{10} form, particularly in terms of the estimated overdispersion parameters and generation times. Cal/2009 R_0 estimates for the threshold functional form are similar to those estimated using the log_{10} functional form, with somewhat greater spread in estimated values. Hong Kong/1968 R_0 estimates for the threshold functional form are somewhat lower than those estimated using the log_{10} functional form. Transmission event parameter estimates for the Hill functional form (Figure F) are very similar to those of the threshold functional form.

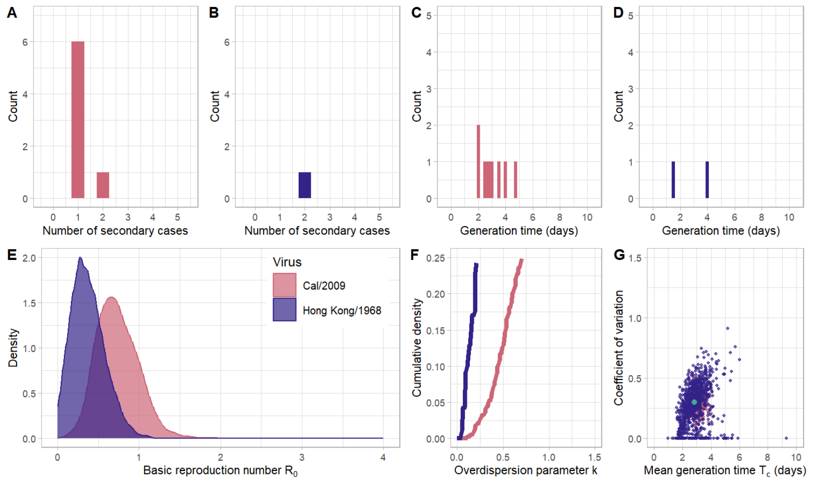

Figure D. Estimation of transmission event parameters for Cal/2009 and Hong Kong/1968, assuming the linear functional form. (A) Distribution of the number of secondary infections generated by n=19 Cal/2009 contact animals. (B) Distribution of the number of secondary infections generated by n=11 Hong Kong/1968 contact animals. In panels (A) and (B), the distributions each show the outcome of one stochastic realization, assuming each animal has 15 one-hour long contacts per day. (C) The generation intervals for each secondary case (n=8) generated in the Cal/2009 simulation shown in panel (A). (D) The generation intervals for each secondary case (n=2) generated in the Hong Kong/1968 simulation shown in panel (B). (E) The distribution of R_0 values from the 1000 Cal/2009 and 1000 Hong Kong/1968 stochastic simulations. (F) The cumulative distribution of overdispersion (k) values from the Cal/2009 and Hong Kong/1968 stochastic simulations that resulted in at least one individual transmitting infection. (G) Estimated means and coefficients of variation of the gamma distributions fit to the generation time distributions of each of the 1000 stochastic simulations for Cal/2009 and each of the 1000 stochastic simulations for Hong Kong/1968. The teal dot indicates the mean and coefficient of variation used to parameterize the gamma distribution that was used to project the intrinsic growth rates in Figure G.

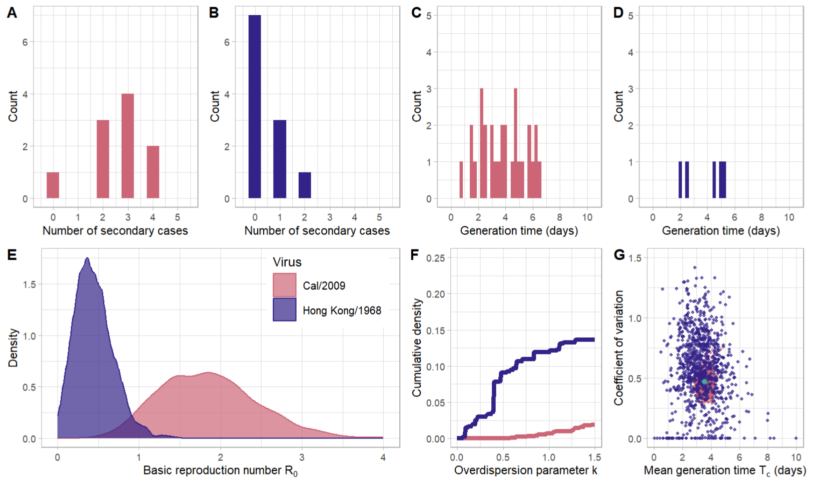

Figure E. Estimation of transmission event parameters for Cal/2009 and Hong Kong/1968, assuming the threshold functional form. (A) Distribution of the number of secondary infections generated by n=19 Cal/2009 contact animals. (B) Distribution of the number of secondary infections generated by n=11 Hong Kong/1968 contact animals. In panels (A) and (B), the distributions each show the outcome of one stochastic realization, assuming each animal has 15 one-hour long contacts per day. (C) The generation intervals for each secondary case (n=26) generated in the Cal/2009 simulation shown in panel (A). (D) The generation intervals for each secondary case (n=5) generated in the Hong Kong/1968 simulation shown in panel (B). (E) The distribution of R_0 values from the 1000 Cal/2009 and 1000 Hong Kong/1968 stochastic simulations. (F) The cumulative distribution of overdispersion (k) values from the Cal/2009 and Hong Kong/1968 stochastic simulations that resulted in at least one individual transmitting infection. (G) Estimated means and coefficients of variation of the gamma distributions fit to the generation time distributions of each of the 1000 stochastic simulations for Cal/2009 and each of the 1000 stochastic simulations for Hong Kong/1968. The teal dot indicates the mean and coefficient of variation used to parameterize the gamma distribution that was used to project the intrinsic growth rates in Figure H.

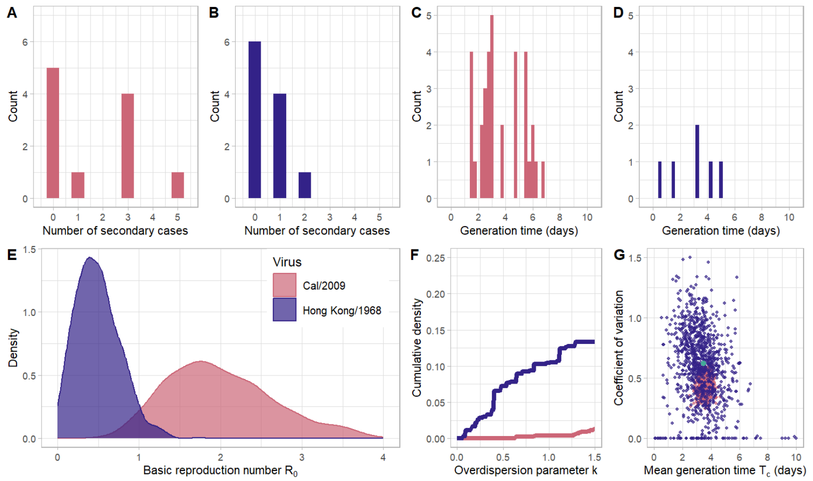

Figure F. Estimation of transmission event parameters for Cal/2009 and Hong Kong/1968, assuming the Hill functional form. (A) Distribution of the number of secondary infections generated by n=19 Cal/2009 contact animals. (B) Distribution of the number of secondary infections generated by n=11 Hong Kong/1968 contact animals. In panels (A) and (B), the distributions each show the outcome of one stochastic realization, assuming each animal has 15 one-hour long contacts per day. (C) The generation intervals for each secondary case (n=18) generated in the Cal/2009 simulation shown in panel (A). (D) The generation intervals for each secondary case (n=5) generated in the Hong Kong/1968 simulation shown in panel (B). (E) The distribution of R_0 values from the 1000 Cal/2009 and 1000 Hong Kong/1968 stochastic simulations. (F) The cumulative distribution of overdispersion (k) values from the Cal/2009 and Hong Kong/1968 stochastic simulations that resulted in at least one individual transmitting infection. (G) Estimated means and coefficients of variation of the gamma distributions fit to the generation time distributions of each of the 1000 stochastic simulations for Cal/2009 and each of the 1000 stochastic simulations for Hong Kong/1968. The teal dot indicates the mean and coefficient of variation used to parameterize the gamma distribution that was used to project the intrinsic growth rates in Figure I.

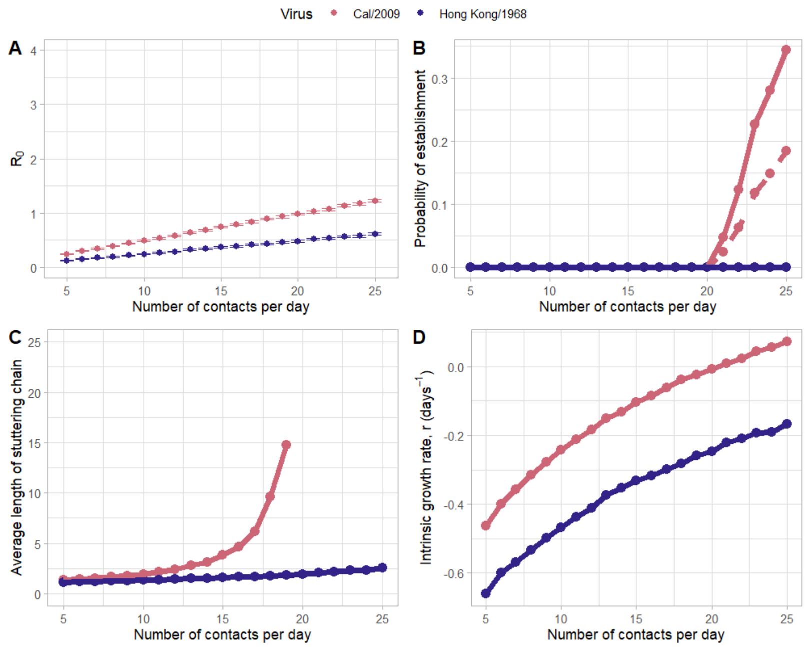

Figure G. Prediction of pandemic potential and dynamics, assuming the linear functional form. (A) Estimates of the reproduction number R_0 for Cal/2009 and Hong Kong/1968 across a range of contact rates. Error bars indicate the 95% confidence intervals. (B) The probability of pandemic establishment for Cal/2009 and Hong Kong/1968 across a range of contact rates. The solid lines indicate probabilities in the absence of transmission heterogeneity ($k = \infty$). The dashed lines indicate probabilities with a moderate level of transmission heterogeneity ($k = 1$). (C) The average length of stuttering transmission chains across the range of contact rates when $R_0 < 1$ for both viruses. (D) Estimated intrinsic growth rates per day for Cal/2009 and Hong Kong/1968 across a range of contact rates, assuming their generation intervals are gamma distributed with mean = 2.85 days and a coefficient of variation of 0.30 (teal point in Figure D).

Prediction of pandemic potential and dynamics for alternative force-of-infection functional forms

In the main text, we predicted pandemic potential and dynamics assuming a log_{10} functional form for the force of infection. Here, we present complementary results for the linear, threshold, and Hill functional forms. The linear functional form (Figure G) generally estimates lower R_0 for both Cal/2009 and Hong Kong/1968, consistent with the results in Figure D. The probability of establishment, length of stuttering transmission chains, and intrinsic growth rate are therefore also lower. The threshold form also estimates somewhat lower R_0 than the log_{10} functional form, though this effect is more pronounced for Hong Kong/1968. This leads to lower estimated probabilities of establishment, length of stuttering transmission chains, and intrinsic growth rates (Figure H). The Hill form results (Figure I) are very similar to those of the threshold form. For all of these additional functional forms, the R_0 estimated for Hong Kong/1968 does not exceed 1 at any of the contact rates tested.

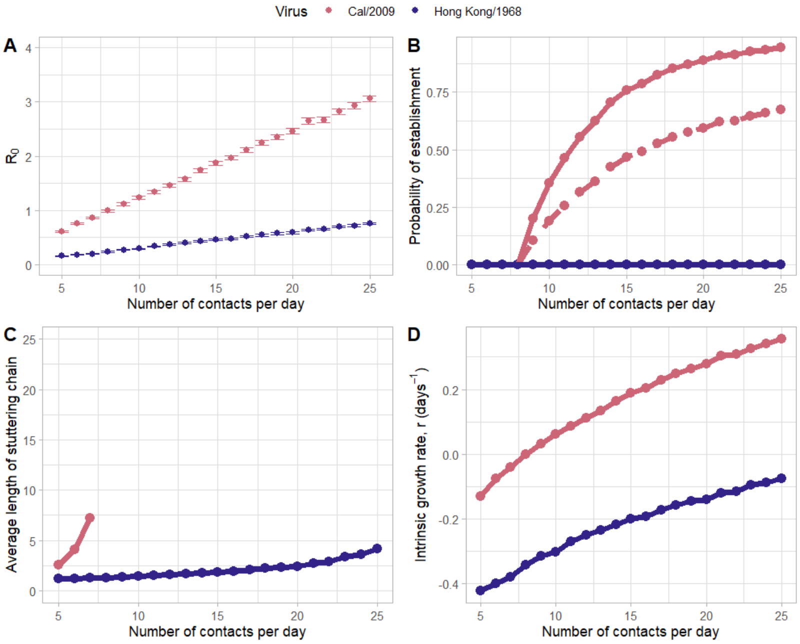

Figure H. Prediction of pandemic potential and dynamics, assuming the threshold functional form. (A) Estimates of the reproduction number R_0 for Cal/2009 and Hong Kong/1968 across a range of contact rates. Error bars indicate the 95% confidence intervals. (B) The probability of pandemic establishment for Cal/2009 and Hong Kong/1968 across a range of contact rates. The solid lines indicate probabilities in the absence of transmission heterogeneity ($k = \infty$). The dashed lines indicate probabilities with a moderate level of transmission heterogeneity ($k = 1$). (C) The average length of stuttering transmission chains across the range of contact rates when $R_0 < 1$ for both viruses. (D) Estimated intrinsic growth rates per day for Cal/2009 and Hong Kong/1968 across a range of contact rates, assuming their generation intervals are gamma distributed with mean = 3.57 days and a coefficient of variation of 0.47 (teal point in Figure E).

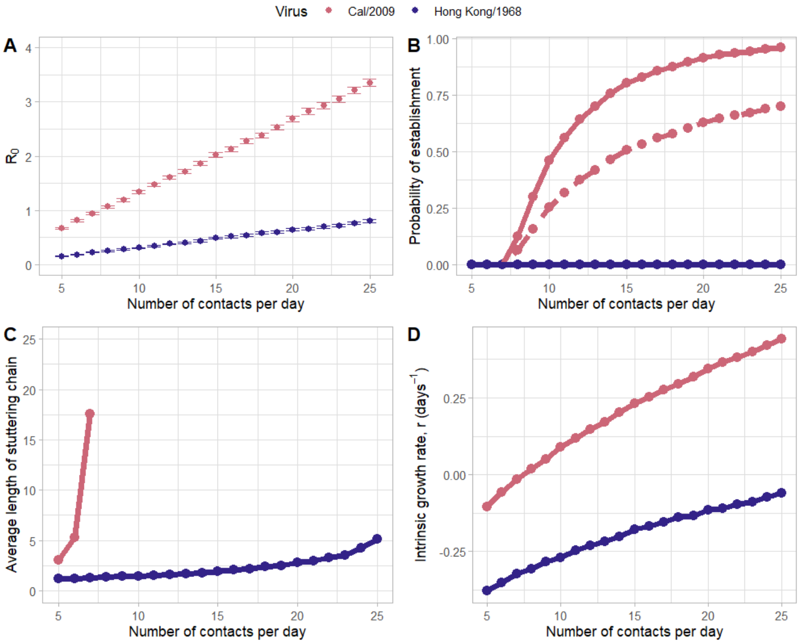

Figure I. Prediction of pandemic potential and dynamics, assuming the Hill functional form. (A) Estimates of the reproduction number R_0 for Cal/2009 and Hong Kong/1968 across a range of contact rates. Error bars indicate the 95% confidence intervals. (B) The probability of pandemic establishment for Cal/2009 and Hong Kong/1968 across a range of contact rates. The solid lines indicate probabilities in the absence of transmission heterogeneity ($k = \infty$). The dashed lines indicate probabilities with a moderate level of transmission heterogeneity ($k = 1$). (C) The average length of stuttering transmission chains across the range of contact rates when $R_0 < 1$ for both viruses. (D) Estimated intrinsic growth rates per day for Cal/2009 and Hong Kong/1968 across a range of contact rates, assuming their generation intervals are gamma distributed with mean = 3.5 days and a coefficient of variation of 0.62 (teal point in Figure F).
