# Supplementary material for: Quantifying viral pandemic potential from experimental transmission studies
Source: PLoS Comput Biol. 2025 Dec 17;21(12):e1013808. doi: 10.1371/journal.pcbi.1013808 (PMC12721528; doi:10.1371/journal.pcbi.1013808)
Supplement: Attachment — Submitted filename: point_by_point.pdf [file pcbi.1013808.s002.pdf]

Dear PLoS Computational Biology, We thank both reviewers for their helpful feedback and the editor for the opportunity to revise and resubmit this work. We have addressed the concerns and suggestions of both reviewers and hope that this revised manuscript (with additional supplemental material) is now suitable for publication in PLoS CB.

With best wishes, Elizabeth Somsen and Katia Koelle

POINT-BY-POINT RESPONSE TO REVIEWER COMMENTS

Note: Line numbers in the responses below refer to those in the pdf with tracked changes. We also have added a supplemental material pdf. This document is entirely new and does not contain tracked changes.

Reviewer: 1

This study proposes a framework for assessing the transmissibility and pandemic potential of viruses using data from animal transmission experiments. First, the viral load and transmission times from these experiments are used to estimate the transmissibility and overall transmission probability for each virus. Then, contacts in a population are simulated to estimate the basic reproduction number for each virus, accounting for overdispersion of viral loads. Finally, by assuming a fixed generation time, the growth rate is estimated for each pathogen. The modelling framework is sound and can be applied widely to animal experiments conducted to estimate pandemic potential, yielding insights beyond counting the proportion of infected animals as is current practice. The code is well documented and runs on the first try.

Comments:

1. It is unclear why different theoretical generation time distributions have been used to calculate the growth rate, rather than the simulated generation time distributions in Figs 4C-4D (averaged over more realisations).

We thank the reviewer for this comment and agree that using the simulated generation time distributions would be preferable. We therefore now fit a gamma distribution to the empirical generation time distribution of each simulation. In Figure 4G, we now instead display the mean values of each of these fit distributions (i.e., the mean generation intervals) against the coefficient of variation of each of these fit distributions. We chose a representative value from these results to use for parameterizing the gamma distribution that then is used to calculate the epidemic growth rate in Figure 5D. We additionally removed the delta and exponential distributions from Figure 5D because the gamma distribution, parameterized from our simulations, is more realistic than either. Appropriate edits have been made in the figures and text to document these changes.

2. Lines 175-177 "Indeed, a qualitative re-inspection of Figure 1 indicates that, for Cal/2009, neither the peak viral nor the duration of infection in contact animals appears to depend on the index's inoculum dose" (and similarly for the other virus) – please conduct a formal statistical analysis as this is an open question in the field.

We thank the reviewer for this suggestion and have conducted the suggested statistical analyses. For Cal/2009, we do not find a positive correlation between peak viral titer

in the contact animal and inoculum dose of its corresponding index animal. For this virus, we also do not find a positive correlation between the duration of infection of the contact animal and inoculum dose of its corresponding index animal. For Hong Kong/1968, viral titer dynamics in the contact animals are more heterogeneous. We do find a positive correlation between peak viral titer in the contact animal and inoculum dose of its corresponding index animal for this virus. For Hong Kong/1968, we find a very weak positive correlation between the duration of infection of a contact animal and the inoculum dose of its corresponding index animal. This weak correlation goes away when reconsidering the duration of infection of two contact animals with transiently positive viral titer measurements. We include these findings in the main text (lines 209-221) and in Figures S1-2.

3. The use of "transmissibility" to refer to the parameter s could be reconsidered. "Transmissibility" commonly refers to the parameter beta in population-level models, and so I would expect this to capture R0 and the transmission probability, rather than only referring to the transmissibility per unit area and then needing to combine "transmissibility" with viral load to get something proportional to R0. However, I recognise that this recommendation is a personal preference.

We thank the reviewer for this comment. We have also struggled with what to call the parameter s and agree that describing it as "transmissibility" will likely cause confusion for the reason that the reviewer notes. Based on this, we have decided to revise text throughout to refer to s only as "parameter s". We have, however, included additional text when introducing this parameter to adequately describe its role in the force-of-infection function (lines 156-159).

4. "In vitro" should be italicised rather than underlined on line 6. Also "a priori" on line 153.

We thank the reviewer for her attention to detail and have now italicized these words.

5. Lines 347-351: "Mutations which increased viral titers in Hong Kong/1968 infected individuals to the level of Cal/2009 would close the gap in transmission potential between these viruses. In contrast, if the primary reason for Cal/2009 having higher transmission potential than Hong Kong/1968 was due to higher transmissibility (higher s), then one might expect that mutations that result in higher transmissibility might be selected for in zoonotic Hong Kong/1968 viral populations." Just because the current gap in R_0 between the viruses is due to differences in viral load, it doesn't mean that this gap could not be closed by mutations that increase s. It is plausible for viruses to reach the same overall R_0 where one has a higher s and lower viral load, and one has a lower s and higher viral load. To make the original argument, one would have to show that the increase in s required is more difficult to attain evolutionarily than the increase in viral load, and it is unclear how to show that.

We thank the reviewer for this comment and completely agree. To address this, we have made text edits to these sentences (lines 436-441).

6. Consider citing Nishiura et al. (2013) PLoS ONE for limitations of existing analyses of ferret transmission data.

We thank the reviewer for pointing us towards this very interesting work and for the suggestion to cite this paper. We now cite this work in the Introduction section (lines

87-94) when discussing the limitations of transmission efficiency outcomes from these types of experiments.

Reviewer: 2

The authors have expanded on an experimental study of influenza A transmission in ferrets. The extension includes data fitting and mathematical modelling to determine the pandemic potential of two different strains.

Comments:

The authors use “transmissibility” to include expelling of virus into the environment, exposure to the virus, and then infection of the secondary subject. Transmission can still occur without infection of the secondary. A clear definition of transmission is needed in this study to understand the method, results and conclusion/discussion. As well, a clear definition of infection is needed.

We thank the reviewer for this comment and have attempted to clarify these terms in the revised manuscript. First, we would like to note that we already have an explicit definition for infection: *We considered an animal to be infected if viral shedding over the limit of detection* (10^1 *TCID50/mL) was observed at any sampled timepoint.* (lines 119-121). Instead of using the term ‘transmissibility’, we now instead explain our approach by relying more heavily, and explicitly, on the concept of force of infection. Finally, we believe that some of the concern here was due to our calling parameter s ’transmissibility s’ (see reviewer 1’s comment). We agree that this was a poor choice and have revised our manuscript accordingly.

There is some discussion of the different viral infection curves between the two strains of the virus. I would like to see some discussion of transmission and infection related to these curves related to high or low viral shedding and large or small sized quanta that the second ferret could be exposed to. Lots of exposure can mean higher viral load at exposure (or even additive exposure over the exposure time window) which can lead to higher probabilities of infection.

We thank the reviewer for this comment, but are having a difficult time interpreting it. At the core of our method is that exposure to higher viral titers over longer periods of time results in higher probabilities of infection. We have attempted to address this concern by adding text to the Discussion section. The text we added discusses another limitation of our approach, namely that the force-of-infection a contact experiences depends on the instantaneous viral titers of the index. We now indicate that the force-of-infection at time t could depend not only on the viral titers at time t but also on the viral titers of the index at times before time t, for example, if it takes time for virions to aggregate into aerosol droplets that are then expelled from the index. These edits to the text can be found on lines 509-515.

Line 111: “As such, viral titer levels appear to impact not only whether a contact animal gets infected but, in the case of infection, when it gets infected.” - please revise this sentence and align ’infection’ with the definitions of ’transmission’ and ’infection’ and some discussion of viral load and quanta.

We thank the reviewer for this suggestion but are of the opinion that this sentence clearly states what we want it to state. We provide the definition of infection in the main text (see also above response).

Line 195 - fix the quotation signs that are in the wrong direction

We thank the reviewer for their attention to detail and have fixed the quotation signs.

The authors have chosen the log10 transmission function. It would be nice to see that other functions in the supplement i.e., related Figures 4 and 5.

We thank the reviewer for this suggestion and have added supplemental figures to show the analogous versions of Figures 4 and 5 for the linear, threshold, and Hill functions. The analogous versions of Figure 4 are provided in Figures S4-6. The analogous versions of Figure 5 are provided in Figures S7-9. We have also added text to the main manuscript, pointing the reader to these supplemental figures.

Paragraph - lines 306-383 - this paragraph is difficult to read. Please revise for flow and length. It could be much shorter and more succinct.

We thank the reviewer for pointing us to areas that they found difficult to read. We have shorted the Discussion section and revised several additional sentences to improve flow.
